# Diaqua­bis­(propane-1,3-diamine)­nickel(II) bis­(propane-1,3-diamine)­disulfato­nickelate(II)

**DOI:** 10.1107/S1600536812009750

**Published:** 2012-03-10

**Authors:** Julia A. Rusanova

**Affiliations:** a National Taras Shevchenko University, Department of Chemistry, Volodymyrska str. 64, 01033 Kyiv, Ukraine

## Abstract

The ionic Ni^II^ title complex, [Ni(C_3_H_10_N_2_)_2_(H_2_O)_2_][Ni(SO_4_)_2_(C_3_H_10_N_2_)_2_], is built up of [Ni(dipr)_2_(H_2_O)_2_]^2+^ complex cations and [Ni(dipr)_2_(SO_4_)_2_]^2−^ complex anions (dipr is propane-1,3-diamine). Both Ni^II^ atoms display a slightly distorted octa­hedral coordination and are located on inversion centers. There are several types of hydrogen-bonding inter­actions, which connect complex cations and anions into a two-dimensional network parallel to (010). Hydrogen bonds formed by the axially coordinated water mol­ecule of the complex cation and one of the O atoms of the sulfate groups of the complex anion (first type) link them into chains along the *c* axis. These chains are linked to each other through hydrogen bonds formed by an O atom (second type) of the SO_4_ groups and NH_2_ groups of the ligand of the complex cations from neighboring chains, forming a two-dimensional hydrogen-bonded net perpendicular to the *b* axis. The third type of O atoms of the sulfate groups of the complex anion are also linked into chains by a combination of both previously described types of H-atom connections.

## Related literature

For background to direct synthesis, see: Nesterov *et al.* (2004 ▶, 2006 ▶); Kovbasyuk *et al.* (1997 ▶, 1998 ▶); Vassilyeva *et al.* (1997 ▶). For the structures of related complexes, see: Clegg *et al.* (1992 ▶); Kim & Lee (2002 ▶); Fritsky *et al.* (2004 ▶); Nowicka *et al.* (2002 ▶); Stockner *et al.* (2007 ▶); Duesler & Raymond (1978 ▶); Jurnak & Raymond (1974 ▶); Solans *et al.* (1982 ▶).
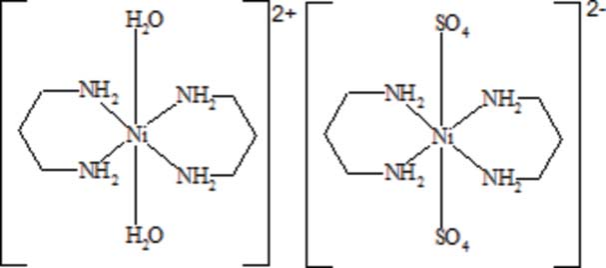


## Experimental

### 

#### Crystal data

[Ni(C_3_H_10_N_2_)_2_(H_2_O)_2_][Ni(SO_4_)_2_(C_3_H_10_N_2_)_2_]
*M* *_r_* = 642.1
Triclinic, 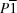

*a* = 6.7055 (1) Å
*b* = 8.9098 (2) Å
*c* = 11.9504 (4) Å
α = 103.016 (2)°
β = 103.795 (2)°
γ = 105.729 (1)°
*V* = 634.52 (3) Å^3^
*Z* = 1
Mo *K*α radiation
μ = 1.71 mm^−1^
*T* = 296 K
0.49 × 0.15 × 0.12 mm

#### Data collection

Bruker APEXII CCD area-detector diffractometer
Absorption correction: numerical (*SADABS*; Sheldrick, 2009 ▶) *T*_min_ = 0.488, *T*_max_ = 0.703
10425 measured reflections
3078 independent reflections
2728 reflections with *I* > 2σ(*I*)
*R*_int_ = 0.030

#### Refinement

*R*[*F* ^2^ > 2σ(*F* ^2^)] = 0.024
*wR*(*F* ^2^) = 0.063
*S* = 1.07
3078 reflections
197 parameters
H atoms treated by a mixture of independent and constrained refinement
Δρ_max_ = 0.41 e Å^−3^
Δρ_min_ = −0.37 e Å^−3^

### 

Data collection: *APEX2* (Bruker, 2007 ▶); cell refinement: *SAINT* (Bruker, 2007 ▶); data reduction: *SAINT*; program(s) used to solve structure: *SHELXTL* (Sheldrick, 2008 ▶); program(s) used to refine structure: *SHELXTL*; molecular graphics: *SHELXTL*; software used to prepare material for publication: *publCIF* (Westrip, 2010 ▶).

## Supplementary Material

Crystal structure: contains datablock(s) I, global. DOI: 10.1107/S1600536812009750/br2192sup1.cif

Structure factors: contains datablock(s) I. DOI: 10.1107/S1600536812009750/br2192Isup2.hkl

Additional supplementary materials:  crystallographic information; 3D view; checkCIF report

## Figures and Tables

**Table 1:** Hydrogen-bond geometry (Å, °)

*D*—H⋯*A*	*D*—H	H⋯*A*	*D*⋯*A*	*D*—H⋯*A*
O5—H9⋯O1	0.82 (2)	1.96 (2)	2.7739 (16)	172 (2)
N2—H1⋯O4	0.823 (19)	2.265 (19)	3.0528 (18)	160.4 (16)
N1—H4⋯O2^i^	0.84 (2)	2.28 (2)	3.0621 (18)	154.9 (18)
O5—H10⋯O2^i^	0.70 (2)	2.15 (3)	2.8476 (18)	179 (3)
N3—H5⋯O3^ii^	0.86 (2)	2.06 (2)	2.8900 (17)	161.4 (17)
N2—H2⋯O2^iii^	0.923 (19)	2.145 (19)	3.0600 (17)	171.2 (15)
N4—H7⋯O3^iv^	0.91 (2)	2.03 (2)	2.9269 (18)	170 (2)

Symmetry codes: (i) 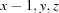; (ii) 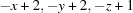; (iii) 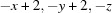; (iv) 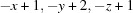.

**Fig. 1.**
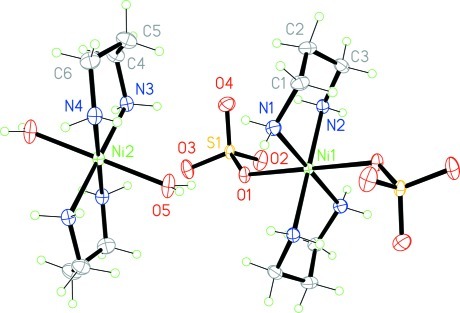
Molecular view of the title compound. Displacement ellipsoids are drawn at the 50% probability level.

**Fig. 2.**
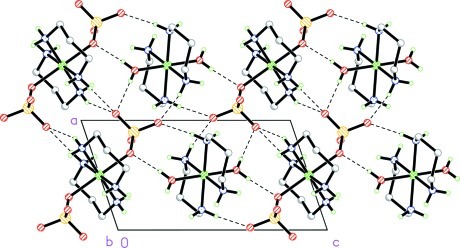
Crystal packing of the title compound. Projection perpendicular to ac-direction. C—H hydrogen atoms are omitted for clarity.

## supplementary crystallographic information

### Comment

It has been shown that direct synthesis is an efficient method to obtained novel homo- and heterometallic complexes (Nesterov *et al.* (2004, 2006); Kovbasyuk *et al.* (1997, 1998); Vassilyeva *et al.* (1997)). The title compound, [Ni(C_3_H_10_N_2_)_2_(H_2_O)_2_][Ni(C_3_H_10_N_2_)_2_(SO_4_)_2_], was obtained unintentionally as the product of an attempted synthesis of a Cu/ Ni mixed-metal complex using zerovalent Copper, Nickel(II) sulfate hexahydrate, diacetylmonoxime and 1,3-diaminopropane in methanol on air. As it shown on Fig. 1. Ni atoms in complex cations and anions display a slightly distorted octahedral coordination, being linked to four N atoms of two 1,3-propane-diamine ligands and two O atoms of two water molecules of complex cation or two sulfo-groups of complex anion.

Three types of hydrogen-bond interactions between O atoms of the sulfogroups and H atoms of the water molecules as well as NH_2_ groups of the 1,3-diaminopropane ligand are presented of Fig 2. The bond distances and angles in the title molecule agree well with the corresponding bond distances and angles reported in closely related compounds (Clegg *et al.*, (1992); Nowicka *et al.*, (2002); Kim & Lee, (2002); Fritsky *et al.*, (2004); Stockner *et al.*, (2007)).

The 1,3-propanediamine ligands have the typical chair conformation and bond distances and angles are similar to those observed elsewhere (Jurnak *et al.*, (1974); Duesler *et al.*, (1978); Solans *et al.*, (1982)).

### Experimental

The title compound was prepared by direct synthesis mixing of the zero valent copper powder (0.064 g, 1 mmol), Ni (SO_4_)_2_^.^H_2_O (0.25 g, 1 mmol), diacetylmonoxime (0.1 g, 1 mmol), 1,3-diaminopropane (0.07 ml, 1 mmol), methanol (20 ml) were heated to 323–333 K and stirred magnetically for 2 h. Resulted mixture was filtered off and transparent brown solution was allowed to stand at room temperature and light blue crystals of the title compound suitable for X-ray analysis precipitated within few days. They were collected by filter-suction, washed with dry Pr^í^OH and finally dried in *vacuo* at room temperature (yield: 0.27 g)

### Refinement

The C—H hydrogen atoms where placed geometrically as riding model, the remaining H atoms were located in difference Fourier synthesis and refined in isotropic approximation.

### Crystal data

**Table:**

[Ni(C_3_H_10_N_2_)_2_(H_2_O)_2_][Ni(SO_4_)_2_(C_3_H_10_N_2_)_2_]	*Z* = 1
*M**_r_* = 642.1	*F*(000) = 340
Triclinic, *P*1	*D*_x_ = 1.680 Mg m^−^^3^
Hall symbol: -P 1	Mo *K*α radiation, λ = 0.71073 Å
*a* = 6.7055 (1) Å	Cell parameters from 5917 reflections
*b* = 8.9098 (2) Å	θ = 2.5–28.2°
*c* = 11.9504 (4) Å	µ = 1.71 mm^−^^1^
α = 103.016 (2)°	*T* = 296 K
β = 103.795 (2)°	Prism, light blue
γ = 105.729 (1)°	0.49 × 0.15 × 0.12 mm
*V* = 634.52 (3) Å^3^	

### Data collection

**Table:**

Bruker APEXII CCD area-detector diffractometer	3078 independent reflections
Radiation source: fine-focus sealed tube	2728 reflections with *I* > 2σ(*I*)
Graphite monochromator	*R*_int_ = 0.030
phi and ω scans	θ_max_ = 28.2°, θ_min_ = 2.5°
Absorption correction: numerical (*SADABS*; Sheldrick, 2009)	*h* = −8→7
*T*_min_ = 0.488, *T*_max_ = 0.703	*k* = −11→10
10425 measured reflections	*l* = −15→15

### Refinement

**Table:**

Refinement on *F*^2^	Primary atom site location: structure-invariant direct methods
Least-squares matrix: full	Secondary atom site location: difference Fourier map
*R*[*F*^2^ > 2σ(*F*^2^)] = 0.024	Hydrogen site location: inferred from neighbouring sites
*wR*(*F*^2^) = 0.063	H atoms treated by a mixture of independent and constrained refinement
*S* = 1.07	*w* = 1/[σ^2^(*F*_o_^2^) + (0.0341*P*)^2^ + 0.0694*P*] where *P* = (*F*_o_^2^ + 2*F*_c_^2^)/3
3078 reflections	(Δ/σ)_max_ = 0.006
197 parameters	Δρ_max_ = 0.41 e Å^−^^3^
0 restraints	Δρ_min_ = −0.37 e Å^−^^3^
12 constraints	

### Special details

**Table:**

Geometry. All e.s.d.'s (except the e.s.d. in the dihedral angle between two l.s. planes) are estimated using the full covariance matrix. The cell e.s.d.'s are taken into account individually in the estimation of e.s.d.'s in distances, angles and torsion angles; correlations between e.s.d.'s in cell parameters are only used when they are defined by crystal symmetry. An approximate (isotropic) treatment of cell e.s.d.'s is used for estimating e.s.d.'s involving l.s. planes.
Refinement. Refinement of *F*^2^ against ALL reflections. The weighted *R*-factor *wR* and goodness of fit *S* are based on *F*^2^, conventional *R*-factors *R* are based on *F*, with *F* set to zero for negative *F*^2^. The threshold expression of *F*^2^ > σ(*F*^2^) is used only for calculating *R*-factors(gt) *etc*. and is not relevant to the choice of reflections for refinement. *R*-factors based on *F*^2^ are statistically about twice as large as those based on *F*, and *R*- factors based on ALL data will be even larger.

### Fractional atomic coordinates and isotropic or equivalent isotropic displacement parameters (Å^2^)

**Table:**

	*x*	*y*	*z*	*U*_iso_*/*U*_eq_	
Ni1	0.5000	1.0000	0.0000	0.01064 (8)	
Ni2	0.5000	1.0000	0.5000	0.01320 (8)	
S1	0.89097 (6)	1.01326 (4)	0.23292 (3)	0.01404 (9)	
O1	0.70097 (16)	1.06228 (13)	0.18226 (9)	0.0155 (2)	
O2	1.05971 (18)	1.06347 (15)	0.17662 (11)	0.0263 (3)	
O3	0.97660 (18)	1.09600 (14)	0.36389 (10)	0.0240 (3)	
O4	0.8164 (2)	0.83460 (14)	0.20784 (11)	0.0287 (3)	
O5	0.4535 (2)	1.07096 (17)	0.33786 (11)	0.0246 (3)	
N1	0.2544 (2)	0.83826 (16)	0.03946 (12)	0.0175 (3)	
N2	0.6059 (2)	0.80081 (15)	−0.05714 (12)	0.0155 (3)	
N3	0.6379 (2)	0.82954 (16)	0.43334 (13)	0.0165 (3)	
N4	0.1851 (2)	0.82026 (17)	0.43194 (13)	0.0180 (3)	
C1	0.1280 (2)	0.67732 (18)	−0.05413 (15)	0.0203 (3)	
H1A	0.0603	0.6943	−0.1294	0.024*	
H1B	0.0126	0.6173	−0.0285	0.024*	
C2	0.2734 (3)	0.57695 (18)	−0.07559 (15)	0.0222 (3)	
H2A	0.1811	0.4660	−0.1253	0.027*	
H2B	0.3524	0.5713	0.0018	0.027*	
C3	0.4379 (3)	0.64240 (18)	−0.13651 (14)	0.0200 (3)	
H3A	0.5084	0.5631	−0.1570	0.024*	
H3B	0.3618	0.6566	−0.2112	0.024*	
C4	0.5382 (3)	0.65484 (19)	0.42206 (15)	0.0226 (3)	
H4A	0.5551	0.6437	0.5024	0.027*	
H4B	0.6151	0.5921	0.3839	0.027*	
C5	0.2966 (3)	0.5844 (2)	0.34818 (16)	0.0264 (4)	
H5A	0.2774	0.6134	0.2738	0.032*	
H5B	0.2489	0.4657	0.3263	0.032*	
C6	0.1537 (3)	0.64477 (19)	0.41474 (16)	0.0246 (4)	
H6A	0.0017	0.5803	0.3696	0.030*	
H6B	0.1856	0.6275	0.4934	0.030*	
H1	0.667 (3)	0.788 (2)	0.0068 (17)	0.018 (5)*	
H2	0.707 (3)	0.830 (2)	−0.0962 (16)	0.018 (4)*	
H3	0.319 (3)	0.826 (2)	0.1079 (18)	0.030 (5)*	
H4	0.165 (3)	0.882 (2)	0.0580 (18)	0.031 (6)*	
H5	0.765 (3)	0.868 (2)	0.4867 (18)	0.023 (5)*	
H6	0.663 (3)	0.836 (2)	0.3633 (18)	0.029 (5)*	
H7	0.127 (3)	0.852 (2)	0.490 (2)	0.037 (6)*	
H8	0.122 (3)	0.828 (2)	0.3723 (18)	0.022 (5)*	
H9	0.533 (4)	1.078 (3)	0.295 (2)	0.041 (6)*	
H10	0.357 (4)	1.070 (3)	0.299 (2)	0.042 (7)*	

### Atomic displacement parameters (Å^2^)

**Table:**

	*U*^11^	*U*^22^	*U*^33^	*U*^12^	*U*^13^	*U*^23^
Ni1	0.00944 (13)	0.01273 (13)	0.01009 (13)	0.00461 (10)	0.00306 (10)	0.00329 (10)
Ni2	0.01046 (13)	0.01788 (14)	0.01234 (14)	0.00583 (10)	0.00410 (10)	0.00503 (10)
S1	0.01275 (17)	0.02077 (18)	0.01136 (17)	0.00832 (14)	0.00423 (14)	0.00661 (14)
O1	0.0124 (5)	0.0248 (5)	0.0122 (5)	0.0096 (4)	0.0042 (4)	0.0067 (4)
O2	0.0192 (6)	0.0456 (7)	0.0277 (6)	0.0188 (5)	0.0154 (5)	0.0192 (6)
O3	0.0206 (6)	0.0370 (6)	0.0124 (5)	0.0131 (5)	0.0019 (5)	0.0036 (5)
O4	0.0374 (7)	0.0228 (6)	0.0256 (6)	0.0128 (5)	0.0043 (6)	0.0099 (5)
O5	0.0158 (6)	0.0495 (8)	0.0182 (6)	0.0175 (6)	0.0084 (5)	0.0176 (5)
N1	0.0169 (6)	0.0183 (6)	0.0196 (7)	0.0065 (5)	0.0085 (6)	0.0067 (5)
N2	0.0158 (6)	0.0186 (6)	0.0140 (6)	0.0085 (5)	0.0051 (5)	0.0052 (5)
N3	0.0129 (6)	0.0215 (6)	0.0152 (7)	0.0070 (5)	0.0037 (6)	0.0052 (5)
N4	0.0143 (6)	0.0239 (7)	0.0164 (7)	0.0073 (5)	0.0040 (6)	0.0070 (5)
C1	0.0156 (7)	0.0162 (7)	0.0260 (8)	0.0015 (6)	0.0050 (7)	0.0071 (6)
C2	0.0254 (8)	0.0139 (7)	0.0264 (9)	0.0059 (6)	0.0076 (7)	0.0059 (6)
C3	0.0225 (8)	0.0172 (7)	0.0182 (8)	0.0082 (6)	0.0054 (7)	0.0012 (6)
C4	0.0214 (8)	0.0214 (8)	0.0251 (8)	0.0108 (6)	0.0052 (7)	0.0052 (6)
C5	0.0225 (8)	0.0209 (8)	0.0279 (9)	0.0056 (6)	0.0027 (7)	0.0005 (7)
C6	0.0181 (8)	0.0224 (8)	0.0299 (9)	0.0033 (6)	0.0062 (7)	0.0079 (7)

### Geometric parameters (Å, º)

**Table:**

Ni1—N1^i^	2.0978 (13)	N2—H2	0.923 (19)
Ni1—N1	2.0978 (13)	N3—C4	1.479 (2)
Ni1—N2^i^	2.1187 (13)	N3—H5	0.86 (2)
Ni1—N2	2.1187 (13)	N3—H6	0.90 (2)
Ni1—O1^i^	2.1257 (10)	N4—C6	1.479 (2)
Ni1—O1	2.1257 (10)	N4—H7	0.91 (2)
Ni2—N3^ii^	2.0893 (13)	N4—H8	0.76 (2)
Ni2—N3	2.0893 (13)	C1—C2	1.518 (2)
Ni2—N4^ii^	2.1083 (13)	C1—H1A	0.9700
Ni2—N4	2.1083 (13)	C1—H1B	0.9700
Ni2—O5	2.1499 (12)	C2—C3	1.521 (2)
Ni2—O5^ii^	2.1499 (12)	C2—H2A	0.9700
S1—O3	1.4645 (11)	C2—H2B	0.9700
S1—O4	1.4682 (12)	C3—H3A	0.9700
S1—O2	1.4688 (11)	C3—H3B	0.9700
S1—O1	1.4945 (10)	C4—C5	1.521 (2)
O5—H9	0.82 (2)	C4—H4A	0.9700
O5—H10	0.70 (2)	C4—H4B	0.9700
N1—C1	1.4806 (19)	C5—C6	1.514 (2)
N1—H3	0.88 (2)	C5—H5A	0.9700
N1—H4	0.84 (2)	C5—H5B	0.9700
N2—C3	1.4785 (19)	C6—H6A	0.9700
N2—H1	0.823 (19)	C6—H6B	0.9700
			
N1^i^—Ni1—N1	180.0	Ni1—N2—H2	109.3 (11)
N1^i^—Ni1—N2^i^	87.81 (5)	H1—N2—H2	109.5 (16)
N1—Ni1—N2^i^	92.19 (5)	C4—N3—Ni2	120.00 (10)
N1^i^—Ni1—N2	92.19 (5)	C4—N3—H5	108.9 (13)
N1—Ni1—N2	87.81 (5)	Ni2—N3—H5	100.4 (12)
N2^i^—Ni1—N2	180.0	C4—N3—H6	108.8 (12)
N1^i^—Ni1—O1^i^	88.28 (5)	Ni2—N3—H6	112.6 (13)
N1—Ni1—O1^i^	91.72 (5)	H5—N3—H6	104.8 (17)
N2^i^—Ni1—O1^i^	92.13 (5)	C6—N4—Ni2	121.41 (10)
N2—Ni1—O1^i^	87.87 (5)	C6—N4—H7	106.6 (13)
N1^i^—Ni1—O1	91.72 (5)	Ni2—N4—H7	101.2 (13)
N1—Ni1—O1	88.28 (5)	C6—N4—H8	107.8 (14)
N2^i^—Ni1—O1	87.87 (5)	Ni2—N4—H8	109.4 (14)
N2—Ni1—O1	92.13 (5)	H7—N4—H8	110 (2)
O1^i^—Ni1—O1	180.00 (6)	N1—C1—C2	111.30 (13)
N3^ii^—Ni2—N3	180.000 (1)	N1—C1—H1A	109.4
N3^ii^—Ni2—N4^ii^	91.66 (5)	C2—C1—H1A	109.4
N3—Ni2—N4^ii^	88.34 (5)	N1—C1—H1B	109.4
N3^ii^—Ni2—N4	88.34 (5)	C2—C1—H1B	109.4
N3—Ni2—N4	91.66 (5)	H1A—C1—H1B	108.0
N4^ii^—Ni2—N4	180.00 (7)	C1—C2—C3	115.06 (13)
N3^ii^—Ni2—O5	87.79 (5)	C1—C2—H2A	108.5
N3—Ni2—O5	92.21 (5)	C3—C2—H2A	108.5
N4^ii^—Ni2—O5	87.84 (5)	C1—C2—H2B	108.5
N4—Ni2—O5	92.16 (5)	C3—C2—H2B	108.5
N3^ii^—Ni2—O5^ii^	92.21 (5)	H2A—C2—H2B	107.5
N3—Ni2—O5^ii^	87.79 (5)	N2—C3—C2	111.60 (13)
N4^ii^—Ni2—O5^ii^	92.16 (5)	N2—C3—H3A	109.3
N4—Ni2—O5^ii^	87.84 (5)	C2—C3—H3A	109.3
O5—Ni2—O5^ii^	180.000 (1)	N2—C3—H3B	109.3
O3—S1—O4	110.23 (7)	C2—C3—H3B	109.3
O3—S1—O2	110.28 (7)	H3A—C3—H3B	108.0
O4—S1—O2	109.99 (7)	N3—C4—C5	112.50 (13)
O3—S1—O1	107.70 (6)	N3—C4—H4A	109.1
O4—S1—O1	109.11 (7)	C5—C4—H4A	109.1
O2—S1—O1	109.48 (6)	N3—C4—H4B	109.1
S1—O1—Ni1	130.04 (6)	C5—C4—H4B	109.1
Ni2—O5—H9	127.9 (16)	H4A—C4—H4B	107.8
Ni2—O5—H10	128.6 (19)	C6—C5—C4	113.29 (14)
H9—O5—H10	101 (2)	C6—C5—H5A	108.9
C1—N1—Ni1	116.97 (10)	C4—C5—H5A	108.9
C1—N1—H3	110.9 (12)	C6—C5—H5B	108.9
Ni1—N1—H3	105.9 (13)	C4—C5—H5B	108.9
C1—N1—H4	108.0 (13)	H5A—C5—H5B	107.7
Ni1—N1—H4	111.9 (13)	N4—C6—C5	113.15 (13)
H3—N1—H4	102.2 (18)	N4—C6—H6A	108.9
C3—N2—Ni1	117.85 (10)	C5—C6—H6A	108.9
C3—N2—H1	108.5 (12)	N4—C6—H6B	108.9
Ni1—N2—H1	103.6 (12)	C5—C6—H6B	108.9
C3—N2—H2	107.8 (11)	H6A—C6—H6B	107.8

Symmetry codes: (i) −*x*+1, −*y*+2, −*z*; (ii) −*x*+1, −*y*+2, −*z*+1.

### Hydrogen-bond geometry (Å, º)

**Table:**

*D*—H···*A*	*D*—H	H···*A*	*D*···*A*	*D*—H···*A*
O5—H9···O1	0.82 (2)	1.96 (2)	2.7739 (16)	172 (2)
N2—H1···O4	0.823 (19)	2.265 (19)	3.0528 (18)	160.4 (16)
N1—H4···O2^iii^	0.84 (2)	2.28 (2)	3.0621 (18)	154.9 (18)
O5—H10···O2^iii^	0.70 (2)	2.15 (3)	2.8476 (18)	179 (3)
N3—H5···O3^iv^	0.86 (2)	2.06 (2)	2.8900 (17)	161.4 (17)
N2—H2···O2^v^	0.923 (19)	2.145 (19)	3.0600 (17)	171.2 (15)
N4—H7···O3^ii^	0.91 (2)	2.03 (2)	2.9269 (18)	170 (2)

Symmetry codes: (ii) −*x*+1, −*y*+2, −*z*+1; (iii) *x*−1, *y*, *z*; (iv) −*x*+2, −*y*+2, −*z*+1; (v) −*x*+2, −*y*+2, −*z*.
